# Efficacy of ceftazidime-avibactam in the treatment of infections due to Carbapenem-resistant *Enterobacteriaceae*

**DOI:** 10.1186/s12879-019-4409-1

**Published:** 2019-09-04

**Authors:** Basem M. Alraddadi, Mohammed Saeedi, Mohammed Qutub, Abeer Alshukairi, Ashraf Hassanien, Ghassan Wali

**Affiliations:** 1 Department of Medicine, King Faisal Specialist Hospital and Research Center, Jeddah, Saudi Arabia; 2 Department of Medicine, University of Jeddah, Jeddah, Saudi Arabia; 3 Department of Pathology and Laboratory Medicine, Section of Clinical Microbiology, King Faisal Specialist Hospital and Research Center, Jeddah, Saudi Arabia; 4 Anti-infectives, Pfizer, Jeddah, Saudi Arabia

**Keywords:** Ceftazidime-avibactam, Carbapenem-resistant *Enterobacteriaceae*, Saudi Arabia

## Abstract

**Background:**

Carbapenem-resistant *Enterobacteriaceae* (CRE) represent an important global threat. The aim of this study is to describe the clinical course and outcomes of patients with CRE infections treated with ceftazidime-avibactam (CAZ-AVI) compared to patients treated with other agents.

**Methods:**

A retrospective cohort study of patients with established CRE infections from January 2017 until August 2018 was conducted. All patients who received CAZ-AVI and all cultures with carbapenem-resistant isolates were screened. We compared patients who received CAZ-AVI for CRE infections with patients who received other agents.

**Results:**

A total of 38 consecutive patients with CRE infections were identified. Age and baseline comorbidities were similar between the two groups. The median time from admission to isolation of CRE culture was 22.5 days in the CAZ-AVI group and 17 days in the comparative group (*P* = 0.7). The incidence of CRE bacteremia was similar between the two groups: 7 patients (70%) in the CAZ-AVI group and 15 patients (53.6%) in the comparative group (*P* = 0.47). The most common type of CRE infections in both groups was hospital acquired pneumonia (HAP). *Klebsiella pneumoniae* was the predominant pathogen in both groups. A carbapenemase gene was detected in 35 (92%) patients; the OXA-48 gene was the predominant gene identified in 28 (74%) isolates. Eight out of ten patients in the CAZ-AVI group and fifteen out of twenty-eight in the comparative group achieved clinical remission (*P* = 0.14). After thirty days, all-cause mortality was observed in five patients in the CAZ-AVI group and 16 patients in the comparative group, accounting for 50 and 57% respectively.

**Conclusions:**

In patients with established OXA-48-type CRE infection, CAZ-AVI is a reasonable alternative to standard therapy. These findings need to be confirmed in prospective studies.

**Electronic supplementary material:**

The online version of this article (10.1186/s12879-019-4409-1) contains supplementary material, which is available to authorized users.

## Background

The emergence of carbapenem-resistant *Enterobacteriaceae* (CRE) represents a threat to global public health [1, 2]. Carbapenems are considered the last line of defence against *Enterobacteriaceae*. The burden of CRE infections is substantial, including longer lengths of stay, higher infection-related mortality and higher health care costs than those associated with carbapenem-susceptible *Enterobacteriaceae* (CSE) [3–5]. Several studies have reported that carbapenem resistance is an independent risk factor for mortality, which is likely due to inappropriate initial antimicrobial therapy [6–8].

Treatment options for CRE infections are very limited. Polymyxins have been used for the treatment of CRE infections; however, there remain concerns regarding increasing resistance, limited efficacy and toxicity [9–12]. Novel β-lactam/β-lactamase inhibitor combinations have emerged as new treatment options for CRE infections [12, 13]. However, these combinations are not active against all carbapenemases [13] . Ceftazidime-avibactam (CAZ-AVI) is a new β-lactam/β-lactamase inhibitor combination with in vitro activity against *Klebsiella pneumoniae* carbapenemases (KPCs) and OXA-48 producing *Enterobacteriaceae* [14]*.*

There is accumulating evidence of the utility of CAZ-AVI for the treatment of infections caused by resistant gram-negative bacilli, including CRE infections [15–19]. However, most of the published studies included patients with KPCs. A recent study documented the successful treatment of patients with OXA-48-type CRE infections with CAZ-AVI [20]. In Saudi Arabia, carbapenemases are highly prevalent in *K pneumoniae* isolates. OXA-48 is the predominant carbapenemase followed by New Delhi metallo- β -lactamase (NDM) [21]. Our aim is to describe the clinical course and outcomes of patients with CRE infections treated with CAZ-AVI compared to those of patients treated with other agents.

## Methods

We conducted a retrospective cohort study of patients with CRE infections from January 2017 to August 2018 at King Faisal Specialist Hospital and Research Center (KFSHRC), Jeddah, Saudi Arabia. All adult patients (> 18 years) who received CAZ-AVI for at least 24 h and all cultures with carbapenem-resistant isolates were screened. We included patients with clinically established CRE infections. Cases where CRE cultures likely represented colonization were excluded. CAZ-AVI was not available at our hospital until December 2017; we compared patients who received CAZ-AVI for the treatment of CRE infections between December 2017 and August 2018 with patients with CRE infections between January 2017 and November 2017 who received other CRE-specific therapies. Baseline characteristics were recorded, and clinical, microbiological and therapeutic data were collected. Clinical course and outcome data until death or hospital discharge were obtained. The primary outcome was complete remission, as evaluated by infectious disease specialists, defined as resolution of fever and eradication of bacteria in subsequent cultures. The secondary outcomes recorded were clinical cure without relapse or death within 30 days, 30-day mortality from starting CAZ-AVI, mortality due to CRE, length of stay in days and 30-day relapse rate with the same isolate.

Data extraction was performed by a trained physician and collected in a special case record form. Data included patients’ demographics, Charlson comorbidity index, concomitant diseases, time from admission to first culture isolate of CRE culture in days, presence of bacteremia, type of infection, organism isolated, time from first CRE culture to starting CRE-specific therapy and CRE-specific therapy used. Susceptibility testing was performed using the Vitek 2 system (bioMérieux, Marcy L’étoile, France) and N-291 card. Phenotypic conformation of CRE was performed using the Clinical Laboratory Standards Institute (CLSI) methodology, which includes the ertapenem, imipenem, and meropenem E-test and modified Hodge test (MHT). All confirmed isolates of CRE from the culture were then tested using the Xpert Carba-R Kit following the manufacture’s recommendation for rapid detection and differentiation of the *bla*_KPC_, *bla*_NDM_, *bla*_VIM_, *bla*_OXA-48_ and *bla*_IMP_ gene sequences linked to carbapenem resistance in gram-negative bacteria. The interpretation of the minimum inhibitory concentration (MIC) for carbapenems is based on CLSI guidelines; resistance to ertapenem was considered if MIC was ≥2 μg/ml and resistance to meropenem and imipenem was considered if MICs were ≥ 4 μg/ml.

All data were analysed using IBM SPP version 25. Continuous data were described using mean and standard deviation for the normally distributed data Medians and interquartile ranges were used for non-normal data. Frequency and percentages were used to describe categorical data. Group comparison was performed using the by chi-square or Fisher’s exact test for proportions. Student’s *t*-test was used to compare continuous data. A *P* value of < 0.05 was considered significant.

## Results

We identified 13 patients who received CAZ-AVI for the treatment of CRE infections between December 2017 and August 2018. Three patients were excluded from the analysis for being children (two patients) and being only treated with only CAZ-AVI for less than 24 h (one patient). The comparative group included 28 patients with CRE infections using the same criteria between January 2017 and November 2017 at KFSHRC-Jeddah. The median age was similar between the two groups: 59.5 years in the CAZ-AVI group and 61.5 years in the comparative group (*P* = 0.71). The median Charlson comorbidity index was 5.5 in the CAZ-AZI group compared to 5 in the comparative group (*P* = 0.86). The demographics and baseline characteristics for the remaining patients are included in Table 1. Both treatment groups were similar, with no significant differences in terms of baseline data. OXA-48 was the predominant carbapenemase in patients who received CAZ-AVI (8/10, 80%), one patient had NDM and in one patient, no carbapenemase gene was detected. In the comparative group, OXA-48 was the predominant carbapenemase as well (19/28, 68%), 5 patients had NDM, one patient had both NDM and OXA-48, and no carbapenemase gene was detected in three patients. Carbapenem MIC distribution was similar between the two groups; details on mechanism of carbapenem resistance and antibiotic MICs for both groups are presented in Additional file 1: Table S1 and Additional file 2: Table S2.

**Table 1:** Baseline characteristics of patients with CRE infections who received ceftazidime/avibactam compared with comparative group (received different CRE specific antibiotics)

Characteristic	Ceftazidime/Avibactam group *n* = 10 (%)	Comparative group *n* = 28 (%)	*P* value
Male	8 (80)	16 (57.1)	0.27
Age, median (IQR), y	59.5 (26–67)	61.5 (50–72)	0.71
CCI, median (IQR)	5.5 (2–8.5)	5 (4–7.75)	0.86
Baseline comorbidities			
Diabetes mellitus	4 (40)	15 (53.6)	0.71
Hypertension	5 (50)	18 (64.3)	0.47
CVD	4 (40)	9 (32.1)	0.71
Renal disease	3 (30)	12 (42.8)	0.71
Malignancy	5 (50)	7 (25)	0.24
Transplant	5 (50)	5 (17.9)	0.09
HIV	0	1 (3.6)	> 0.99
Time from admission to first isolate of CRE culture (days), median (IQR), days	22.5 (4.75–50.75)	17 (5.25–29.25)	0.71
CRE Bacteremia	7 (70)	15 (53.6)	0.47
Type of infection			
CLABSI	1 (10)	4 (14.3)	> 0.99
HAP	5 (50)	14 (50)	> 0.99
cUTI	3 (30)	8 (28.6)	> 0.99
cIAI	3 (30)	5 (17.8)	0.41
SSTI	2 (20)	3 (10.7)	0.59
Microbiology			
*Klebsiella pneumoniae*	7 (70)	23 (82.1)	0.41
*Escherichia coli*	3 (30)	5 (17.9)	
Time from first CRE culture to starting CRE specific therapy, median (IQR), days	3.5 (1–8.75)	0 (0–1)	0.05

*IQR* Interquartile range, *CCI* Charlson comorbidity index, *CVD* Cardiovascular disease, *HIV* Human immunodeficiency virus, *CLABSI* Central Line-associated blood stream infection, *CRE* Carbapenem-resistant *Enterobacteriaceae*, *HAP* Hospital-acquired pneumonia, *cUTI* Chronic urinary tract infection, *cIAI* complicated intra-abdominal infection, *SSTI* Soft tissue infection

The median time (IQR) to the first isolation of CRE was 22.5 days (4.75–50.75) in the CAZ-AVI group compared to 17 days (5.25–29.25) in the comparative group. Hospital-acquired pneumonia (HAP) was the most common infection type in both groups, with 5 (50%) patients in the CAZ-AVI group compared to 14 (50%) in the comparative group (> 0.99). *K. pneumoniae* was the predominant pathogen in both groups. Details of baseline characteristics of both groups are included in Table 1. Groups remained similar after restricting analysis on patients with OXA-48 carbapenemase gene (Additional file 3: Table S3).

The comparative group received colistin (21, 75%), carbapenem (21, 75%), tigecycline (9, 32.1%), aminoglycoside (8, 28.6%), quinolone (4, 14.3%), trimethoprim/sulfamethoxazole (1, 3.6%) and aztreonam (1, 3.6%). Out of the 28 patients in the comparative group, 25 patients were administered antibiotic combinations. Details of the used combinations in the comparative group are presented in Additional file 4: Table S4.

Eight patients (80%) in the CAZ-AVI group achieved clinical remission compared to 15 patients (53.6%) in the comparative group (*P* = 0.14). The time to clearance of bacteremia was similar in both groups, with median (IQR) values of 4 days (3–5) and 5 days (3–7), respectively (Table 2). Thirty-day all-cause mortality and relapse with the same isolate were similar between the two groups (5, 50%) vs (16, 57.1%) (*P* = 0.7) and (2, 20%) vs (1, 3.6%) (*P* = 0.1) respectively. Other secondary outcomes including relapse, attributable mortality and 30-day mortality, were similar in both groups. There was no difference in outcome results after restricting analysis on patients with OXA-48 carbapenemase gene (Additional file 5: Table S5).

**Table 2:** Outcomes of patients with CRE infections who received ceftazidime-avibactam compared with comparative group (received different CRE specific antibiotics)

Outcome	Ceftazidime/Avibactam group *n* = 10 (%)	Comparative group *n* = 28 (%)	*P* value
Clinical remission	8 (80)	15 (53.6)	0.14
Clinical cure without relapse or	4 (40)	11 (39)	> 0.99
death within 30 days			
30 days all-cause mortality	5 (50)	16 (57.1)	0.7
Attributable mortality to CRE	2 (20)	11 (39.3)	0.27
Length of stay, median (IQR), days	69.5 (47.5–96)	40.5 (22–79.5)	0.07
30-days relapse of the same isolate	2 (20)	1 (3.6)	0.1
Time to clearance of bacteremia,	4 (3–5)	5 (3–7)	0.65
median (IQR), days			

*CRE* Carbapenem-resistant *Enterobacteriaceae*, *IQR* Interquartile range

## Discussion

Even though our small sample size likely precluded our ability to find statistically significant differences, our study demonstrated a clinically significant benefit of CAZ-AVI for the treatment of CRE infections, including those caused by OXA-48 producing organisms, compared to standard therapy. A majority of our patients had HAP infections. All-cause mortality in previously reported studies ranged from 24 to 39.5% compared to 50–57.1% in our cohort; we hypothesize that this difference is due to the high comorbidity index in our patients, reflecting the complex medical background and severe nature of these infections.

Many studies have investigated the role of various combination therapies for the treatment of CRE; two large retrospective studies showed that combination therapy (with two or more in vitro-active drugs, with meropenem in all patients) was associated with lower mortality rates than monotherapy (colisin, tigecycline, and gentamicin) in populations with high severity indices [22, 23].

CAZ-AVI has emerged as a promising therapy for CRE infections in several clinical studies, however, most of these studies included patients with KPCs [17, 24]. In a prospective multicenter cohort study to describe the clinical outcomes for patients with CRE infections compared, 38 patients treated with CAZ-AVI to 99 patients treated with colistin for KPC-producing CRE, it showed lower adjusted all-cause mortality in the CAZ-AVI group [24]. Sousa A. et al prospectively studied the effectiveness of CAZ-AVI as a rescue treatment for managing infections due to OXA-48-producing *Enterobacteriaceae* in 57 patients, 81% received CAZ-AVI as a monotherapy [20].

New treatment options for CRE infections have emerged in recent years, many of which exhibit activity against the KPC-producing gene but not OXA-48. Meropenem/vaborbactam is a novel β -lactamase inhibitor that exert activity against CRE by inhibiting class A carbapenemases such as KPCs but has no in vitro activity against class B metallo- β-lactamases (NDM or VIM) or class D OXA 48 B-lactamases [25]. Plazomicin is a next-generation aminoglycoside that has been approved for the treatment of complicated urinary tract infections (cUTI). Studies have shown that plazomicin is more potent than other aminoglycosides against KPC-producing *Enterobacteriaceae* [12]. Eravasycline is a synthetic antibacterial agent of the tetracycline class that has been approved for the treatment of complicated intra-abdominal infections (cIAI) and has been shown to have twofold higher activity than tigecycline against CRE; however, there is limited clinical data for the efficacy of this drug against CRE infections [12]. Imipenem/cilastin and relebactam is a combination of imipenem and a novel β -lactamase inhibitor that exert activity against bacteria by inhibiting class A and C carbapenemases but has no in vitro activity against class B metallo- β -lactamases or class D OXA-48 B-lactamasese. Cefiderocol is a novel cephalosporin with unique antibacterial activity against many CRE isolates and is active against carbapenemase hydrolysis [26].

The most prevalent carbapenemase-producing gene in Saudi Arabia is OXA-48, followed by NDM. In our cohort, a majority of the tested isolates carried the OXA-48 gene [21]. Because of the different performances of these antimicrobial agents, we strongly stress on the importance of molecular testing to identify the gene responsible for causing the CRE infection and thus use the appropriate antibiotic.

The emergence of CAZ-AVI-resistant strains during treatment has already been reported and can be a contributing factor to increased mortality among patients with OXA-48 type CRE infections [16]. Shields et al described resistance to CAZ-AVI in 3 out of 10 patients with microbiological failure following treatment for 10–19 days. We believe that CAZ-AVI should be incorporated in standard antibiogram susceptibility testing.

Our study has several limitations. First, the retrospective nature of the analysis is a source of confounding by the indication type of bias. Additionally, due to the small sample size, our study was not sufficiently statistically powerful to detect a significant difference in efficacy or tolerability. Randomized controlled trials to address this vital issue are needed.

## Conclusions

In summary, our study included a cohort of patients with invasive CRE infections, a majority of whom exhibited OXA-48 genotype, and showed that CAZ-AVI is a promising antibiotic for the treatment of these patients with limited therapeutic options. Despite the limitations and small size of the study, we were able to show that CAZ-AVI is effective and comparable to standard treatment for patients with established OXA-48-type CRE infections.

## Additional files

Additional file 1: **Table S1.** Mechanism of carbapenem resistance and minimum inhibitory concentration for ceftazidime-avibactam group. (DOC 40 kb)

Additional file 2: **Table S2.** Mechanism of carbapenem resistance and minimum inhibitory concentration for the comparative group. (DOCX 15 kb)

Additional file 3: **Table S3.** Baseline characteristics of patients with OXA-48 CRE infections who received ceftazidime/avibactam compared with comparative group (received different CRE specific antibiotics) (DOC 51 kb)

Additional file 4: **Table S4.** Frequency of antibiotic combinations used for treatment of CRE infections in the comparative group. (DOCX 16 kb)

Additional file 5: **Table S5.** Outcomes of patients with OXA-48 CRE infections who received ceftazidime-avibactam compared with comparative group (received different CRE specific antibiotics). (DOC 41 kb)

## Data Availability

The datasets used and / or analyzed during the current study are available from the corresponding author on reasonable request.

## Abbreviations

CAZ-AVI: Ceftazidime-Avibactam
cIAI: Complicated intra-abdominal infections
CLSI: Clinical Laboratory Standards Institute
CRE: Carbapenem-resistant *Enterobacteriaceae*
CSE: Carbapenem-susceptible *Enterobacteriaceae*
cUTI: Complicated urinary tract infections
HAP: Hospital acquired pneumonia
IQR: Interquartile range
KPC: *Klebsiella pneumoniae* carbapenamase
MHT: Modified Hodge test
MIC: Minimum inhibitory concentration
NDM: New Delhi metallo- β -lactamase
