# Supplementary material for: Efficacy of ceftazidime-avibactam in the treatment of infections due to Carbapenem-resistant Enterobacteriaceae
Source: BMC Infect Dis. 2019 Sep 4;19:772. doi: 10.1186/s12879-019-4409-1 (PMC6724371; doi:10.1186/s12879-019-4409-1)
Supplement: Supplementary file 1 — Table S1. Mechanism of carbapenem resistance and minimum inhibitory concentration for ceftazidime-avibactam group. (DOC 40 kb) [file 12879_2019_4409_MOESM1_ESM.doc]

Table S1: Mechanism of carbapenem resistance and minimum inhibitory concentration for ceftazidime-avibactam group

| Patient number | Organism | Mechanism of carbapenem resistance | Ceftazidime-Avibactam  MIC (μg/ml) | Meropenem  MIC (μg/ml) | Imipenem  MIC (μg/ml) |
| --- | --- | --- | --- | --- | --- |
| 1 | *Klebsiella pneumoniae* | OXA-48 | 2 | 32 | 32 |
| 2 | *Klebsiella pneumoniae* | OXA-48 | 2 | 32 | 32 |
| 3 | *Escherichia coli* | NDM | 256 | 32 | 32 |
| 4 | *Klebsiella pneumoniae* | OXA-48 | NA | 32 | 32 |
| 5 | *Klebsiella pneumoniae* | Not Detected | NA | 4 | 8 |
| 6 | *Klebsiella pneumoniae* | OXA-48 | 4 | 32 | 32 |
| 7 | *Escherichia coli* | OXA-48 | 0.50 | 16 | 16 |
| 8 | *Klebsiella pneumoniae* | OXA-48 | 2 | 32 | 32 |
| 9 | *Klebsiella pneumoniae* | OXA-48 | 4 | 32 | 16 |
| 10 | *Escherichia coli* | OXA-48 | NA | 16 | 32 |

MIC minimum inhibitory concentration; NA, Not available
