# Supplementary material for: Efficacy of ceftazidime-avibactam in the treatment of infections due to Carbapenem-resistant Enterobacteriaceae
Source: BMC Infect Dis. 2019 Sep 4;19:772. doi: 10.1186/s12879-019-4409-1 (PMC6724371; doi:10.1186/s12879-019-4409-1)
Supplement: Supplementary file 2 — Table S2. Mechanism of carbapenem resistance and minimum inhibitory concentration for the comparative group. (DOCX 15 kb) [file 12879_2019_4409_MOESM2_ESM.docx]

Table S2: Mechanism of carbapenem resistance and minimum inhibitory concentration for the comparative group

| Patient number | Organism | Mechanism of carbapenem resistance | Meropenem  MIC (μg/ml) | Imipenem  MIC (μg/ml) |
| --- | --- | --- | --- | --- |
| 1 | *Escherichia coli* | Not Detected | 2 | 0.5 |
| 2 | *Klebsiella pneumoniae* | OXA-48 | 4 | 4 |
| 3 | *Klebsiella pneumoniae* | OXA-48 | 32 | 32 |
| 4 | *Escherichia coli* | NDM | 32 | 32 |
| 5 | *Klebsiella pneumoniae* | NDM | 16 | 16 |
| 6 | *Escherichia coli* | NDM | 32 | 32 |
| 7 | *Escherichia coli* | OXA-48 | 16 | 16 |
| 8 | *Klebsiella pneumoniae* | OXA-48 | 16 | 16 |
| 9 | *Klebsiella pneumoniae* | NDM and OXA-48 | 16 | 16 |
| 10 | *Klebsiella pneumoniae* | OXA-48 | 32 | 8 |
| 11 | *Klebsiella pneumoniae* | NDM | 16 | 16 |
| 12 | *Klebsiella pneumoniae* | OXA-48 | 16 | 8 |
| 13 | *Klebsiella pneumoniae* | OXA-48 | 32 | 8 |
| 14 | *Klebsiella pneumoniae* | NDM | 16 | 16 |
| 15 | *Klebsiella pneumoniae* | Not Detected | 16 | 16 |
| 16 | *Klebsiella pneumoniae* | OXA-48 | 16 | 2 |
| 17 | *Klebsiella pneumoniae* | OXA-48 | 8 | 4 |
| 18 | *Klebsiella pneumoniae* | OXA-48 | 16 | 4 |
| 19 | *Klebsiella pneumoniae* | OXA-48 | 16 | 32 |
| 20 | *Klebsiella pneumoniae* | OXA-48 | 32 | 32 |
| 21 | *Klebsiella pneumoniae* | OXA-48 | 8 | 4 |
| 22 | *Klebsiella pneumoniae* | OXA-48 | 32 | 32 |
| 23 | *Klebsiella pneumoniae* | OXA-48 | 16 | 2 |
| 24 | *Klebsiella pneumoniae* | NA | 32 | 32 |
| 25 | *Klebsiella pneumoniae* | OXA-48 | 16 | 4 |
| 26 | *Klebsiella pneumoniae* | OXA-48 | 32 | 32 |
| 27 | *Klebsiella pneumoniae* | OXA-48 | 32 | 32 |
| 28 | *Klebsiella pneumoniae* | OXA-48 | 4 | 16 |

MIC minimum inhibitory concentration; NA, Not available
