# Supplementary material for: Efficacy of ceftazidime-avibactam in the treatment of infections due to Carbapenem-resistant Enterobacteriaceae
Source: BMC Infect Dis. 2019 Sep 4;19:772. doi: 10.1186/s12879-019-4409-1 (PMC6724371; doi:10.1186/s12879-019-4409-1)
Supplement: Supplementary file 3 — Table S3. Baseline characteristics of patients with OXA-48 CRE infections who received ceftazidime/avibactam compared with comparative group (received different CRE specific antibiotics) (DOC 51 kb) [file 12879_2019_4409_MOESM3_ESM.doc]

Table S3: Baseline characteristics of patients with OXA-48 CRE infections who received ceftazidime/avibactam compared with comparative group (received different CRE specific antibiotics)

| Characteristic | Ceftazidime/Avibactam group *n*=8 (%) | Comparative group  *n*=20 (%) | *P* value |
| --- | --- | --- | --- |
| Male | 8 (87.5) | 13 (65) | 0.37 |
| Age, median (IQR), y | 59.5 (30.25 – 67) | 62.5 (54.75 – 72) | 0.18 |
| CCI, median (IQR) | 5.5 (2.5 – 8) | 6 (5 – 8) | 0.6 |
| Baseline comorbidities | | | |
| Diabetes mellitus | 3 (37.5) | 12 (60) | 0.41 |
| Hypertension | 4 (50) | 13 (65) | 0.67 |
| CVD | 3 (37.5) | 8 (40) | >0.99 |
| Renal disease | 2 (25) | 11 (55) | 0.22 |
| Malignancy | 4 (50) | 5 (25) | 0.2 |
| Transplant | 4 (50) | 1 (5) | 0.15 |
| HIV | 0 | 1 (5) | 0.71 |
| Time from admission to first isolate of CRE culture (days), median (IQR), days | 18 (4.25 – 25.5) | 18.5 (9.5 – 29.25) | 0.94 |
| CRE Bacteremia | 7 (87.5) | 13 (65) | 0.37 |
| Type of infection | | | |
| CLABSI | 1 (12.5) | 2 (10) | >0.99 |
| HAP | 4 (50) | 11 (55) | >0.99 |
| cUTI | 2 (25) | 5 (25) | >0.99 |
| cIAI | 3 (37.5) | 3 (15) | 0.311 |
| SSTI | 1 (12.5) | 2 (10) | >0.99 |
| Microbiology | | | |
| *Klebsiella pneumoniae* | 6 (75) | 18 (90) | 0.56 |
| *Escherichia coli* | 2 (25) | 2 (10) |  |
| Time from first CRE culture to starting CRE specific therapy, median (IQR), days | 3.5 (1 – 8) | 0 (0 – 1) | 0.04 |
| IQR, interquartile range; CCI, Charlson comorbidity index; CVD, cardiovascular disease; HIV, human immunodeficiency virus; CLABSI, central Line-associated blood stream infection; CRE, carbapenem-resistant *Enterobacteriaceae*; HAP, hospital-acquired pneumonia; cUTI, chronic urinary tract infection; cIAI, complicated intra-abdominal infection; SSTI, soft tissue infection. | | | |
