# Supplementary material for: Efficacy of ceftazidime-avibactam in the treatment of infections due to Carbapenem-resistant Enterobacteriaceae
Source: BMC Infect Dis. 2019 Sep 4;19:772. doi: 10.1186/s12879-019-4409-1 (PMC6724371; doi:10.1186/s12879-019-4409-1)
Supplement: Supplementary file 4 — Table S4. Frequency of antibiotic combinations used for treatment of CRE infections in the comparative group. (DOCX 16 kb) [file 12879_2019_4409_MOESM4_ESM.docx]

Table S4: Frequency of antibiotic combinations used for treatment of CRE infections in the comparative group

| Antibiotics combination | *n (%)* |
| --- | --- |
| Colistin/Carbapenem | 7 (25%) |
| Colistin/Carbapenem/aminoglycoside | 5(17.8%) |
| Colistin/Carbapenem/tigecycline | 2 (7.1%) |
| Colistin/tigecycline | 2 (7.1%) |
| Carbapenem/tigecycline | 2 (7.1%) |
| Colistin/Carbapenem/quinolone | 1 (3.6%) |
| Colistin/Carbapenem/trimethoprim/sulfamethoxazole | 1 (3.6%) |
| Colistin/Carbapenem/aztreonam | 1 (3.6%) |
| Colistin/tigecycline/aminoglycoside | 1 (3.6%) |
| Carbapenem/quinolone | 1 (3.6%) |
| Colistin/tigecycline/aminoglycoside | 1 (3.6%) |
| Quinolone/aminoglycoside | 1 (3.6) |
