# Supplementary material for: Efficacy of ceftazidime-avibactam in the treatment of infections due to Carbapenem-resistant Enterobacteriaceae
Source: BMC Infect Dis. 2019 Sep 4;19:772. doi: 10.1186/s12879-019-4409-1 (PMC6724371; doi:10.1186/s12879-019-4409-1)
Supplement: Supplementary file 5 — Table S5. Outcomes of patients with OXA-48 CRE infections who received ceftazidime-avibactam compared with comparative group (received different CRE specific antibiotics). (DOC 41 kb) [file 12879_2019_4409_MOESM5_ESM.doc]

Table S5: Outcomes of patients with OXA-48 CRE infections who received ceftazidime-avibactam compared with comparative group (received different CRE specific antibiotics)

| Outcome | Ceftazidime/Avibactam | Comparative group | *P* value |
| --- | --- | --- | --- |
|  | group *n*=8 (%) | *n*=20 (%) |  |
| Clinical remission | 6 (75) | 8 (40) | 0.21 |
| Clinical cure without relapse or | 3 (37.5) | 8 (40) | >0.99 |
| death within 30 days | | | |
| 30 days all-cause mortality | 3 (37.5) | 10 (50) | 0.69 |
| Attributable mortality to CRE | 2 (25) | 10 (50) | 0.40 |
| Length of stay, median (IQR), days | 62 (43.5 – 88.25) | 40.5 (21.25 – 79.5) | 0.5 |
| 30-days relapse of the same isolate | 2 (20) | 0 | 0.74 |
| Time to clearance of bacteremia, | 4.5 (2.75-5.25) | 4 (2 – 6) | 0.32 |
| median (IQR), days | | | |

CRE, carbapenem-resistant *Enterobacteriaceae*; IQR, interquartile range.
